# Inferring Geographic Origin of White‐Tailed Deer (*Odocoileus virginianus*) Using Single Nucleotide Polymorphisms in a Region Experiencing Long‐Term Effects of Chronic Wasting Disease

**DOI:** 10.1002/ece3.74454

**Published:** 2026-10-09

**Authors:** Alberto F. Fameli, Jessie E. Edson, Krysten L. Schuler, W. David Walter

**Affiliations:** ^1^ Department of Ecosystem Science and Management The Pennsylvania State University University Park Pennsylvania USA; ^2^ Veterinary Microbiology and Pathology Washington State University Pullman Washington USA; ^3^ Cornell Wildlife Health Lab New York State Wildlife Health Program Ithaca New York USA

**Keywords:** assignment tests, chronic wasting disease, disease management, genetic structure, *Odocoileus virginianus*, origin inference

## Abstract

Chronic wasting disease (CWD) is a contagious, 100% fatal disease affecting North American cervids. White‐tailed deer ( *Odocoileus virginianus* ) is the most abundant host, and movements of this species, either natural or human‐mediated, have been considered primary drivers of CWD spread. Inferring the geographic origin of CWD‐positive individuals can help identify source–sink dynamics of disease spread and anthropogenic translocation of carcasses. We explored two approaches to infer origin of wild white‐tailed deer: (1) a combination of genetic structure inference and categorical assignment tests, and (2) continuous assignment methods (SPA and LOCATOR) to infer coordinates of origin, based on comparisons between test and reference genotypes. We analyzed 1749 samples genotyped using a recently developed single nucleotide polymorphism panel, retaining > 63,000 markers. Genetic structure analyses identified four, five, or ten clusters, with barriers to gene flow that were consistent between the different analyses and explainable by landscape features. We used the ten‐cluster configuration to perform categorical assignment tests, but these tests showed low–moderate performance (assignment accuracy ranged between 41% and 100% among clusters, average was 71%). SPA produced a high level of inaccuracy in inferred origins. In contrast, LOCATOR assigned most individuals (82%) to an origin < 50 km from their sampling coordinates. Given the species' life history traits (e.g., vagility, overlapping generations, polygamy), this level of accuracy suggests that continuous assignment tests may be a valuable tool for wildlife managers. Additionally, the inferred genetic structure can inform the delineation of management units, with implications for population control and mitigation of contagious diseases.

## Graphical abstract

We analyzed 1749 samples of white‐tailed deer ( *Odocoileus virginianus* ) genotyped for > 63,000 single nucleotide polymorphisms to assess genetic structure in the mid‐Atlantic region of the USA, and to evaluate the effectiveness of genetic methods to retrieve the correct geographic origin of deer. The inferred genetic structure can inform the delineation of management units, with implications for population control and mitigation of contagious diseases. Continuous genetic assignments placed most individuals (82%) into an origin < 50 km from their sampling coordinates, which, given the species' life history traits, represents a level of accuracy that may be valuable for wildlife managers in detecting human‐mediated movement of deer.

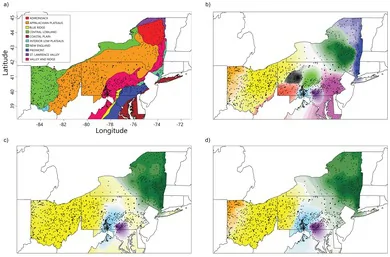

## 1 Introduction

Wildlife management requires defining units that will be subject to different regulations, monitoring activities, or actions meant to control infectious diseases. The delineation of these management units can sometimes incorporate biological information, such as telemetry data to assess barriers in animal movement, but in many cases the borders are drawn following administrative boundaries, topographic features, infrastructure, or public input (VerCauteren and Hygnstrom 2011; Bischof et al. 2016; Florida Fish and Wildlife Conservation Commission 2026). In these cases, management units are unlikely to reflect real biological structure, for example by partitioning one biological group into multiple small management units. In cases like this, there is a potential for animals to move from one management unit to another, creating errors in monitoring assessments. Genetic analyses can be used to elucidate the presence of genetic clusters that are more or less isolated and behave as independent demographic units. As egression and ingression of animals is expected to be infrequent in each cluster, hence the maintenance of a separate genetic identity, these clusters can be used as guides for delineating management units.

Ungulates occupy a predominant place in wildlife management conducted in the United States of America (Krausman and Bleich 2013). In contemporary management of ungulates, particularly of deer, there is an emphasis to control chronic wasting disease (CWD). Since its first detection almost 60 years ago in a captive facility in Colorado, USA (Williams and Young 1980), CWD has spread to numerous wild populations. To this day, it has been detected in free‐ranging cervids from 37 states, four Canadian provinces, and three Scandinavian countries (Benestad et al. 2016; Hopp et al. 2024; U.S. Geological Survey 2026). It remains the only self‐sustaining prion disease affecting wildlife (Angers et al. 2009), and like the other known transmissible spongiform encephalopathies, it is invariably fatal (Prusiner 1998). While the risk that CWD poses to human health is speculative, its effects on the cervid populations it infects can be potentially devastating (DeVivo et al. 2017; Edmunds et al. 2016; Monello et al. 2014).

White‐tailed deer ( *Odocoileus virginianus* ) is the most common deer species in North America, and the primary species affected by CWD (Crête and Daigle 1999; VerCauteren 2003; Heffelfinger 2011; Miller et al. 2020). Therefore, white‐tailed deer movements are likely to be the main driver of natural CWD spread into new areas. This species also has a predominant economic value, being the most harvested species in the United States of America (Readyhough et al. 2025).

In wild cervid populations, CWD management is conducted through population reduction either by government agencies or public hunting (Williams and Miller 2002; Wasserberg et al. 2009; Manjerovic et al. 2014; Potapov et al. 2016; Schuler et al. 2022). Additionally, intense surveillance efforts (Evans et al. 2014; Joly et al. 2009) and restrictions on the movement of deer parts from CWD areas have been implemented to slow down the spread into new areas (Gagnier et al. 2020). In this context, the ability to determine the origin of CWD‐positive deer found in the wild can be crucial to the effectiveness of disease management and response. Cases where the location of a CWD‐infected deer and its inferred origin are too far from each other to be explained by natural dispersion would warrant further investigation.

Genetic assignment tests are used to infer the origin of individuals by comparing them to a reference sample, making these tests essentially different from clustering analyses where the goal is to identify potential genetic clusters using all individuals in the sample. Assignment tests have been successfully applied to determine captive or wild origin of white‐tailed deer, using microsatellite markers and samples from the mid‐Atlantic region (Pennsylvania, Maryland, and Virginia) of the USA (Miller and Walter 2020). This was achieved by implementing categorical assignment tests, which are limited to placing individuals into predefined groups based on genetic similarity. In another study using the same methodology, wild deer from a larger region of the mid‐Atlantic were assigned to nine genetic subpopulations previously inferred via genetic clustering analysis (Walter et al. 2024), as a way to test the efficiency of categorical assignments in retrieving the correct geographic origin of individuals. In this case, the assignments showed low accuracy, with correct assignment of deer to the nine subpopulations ranging from 51% to 82%. This could indicate a degree of genetic differentiation between subpopulations too low to be useful for categorical assignment tests, but high enough for the genetic clustering analysis to identify separate groups.

The recent development of a panel of single nucleotide polymorphisms (SNPs) for white‐tailed deer (Navarro et al. 2026) has created the possibility of analyzing thousands of genetic markers instead of the commonly used < 20 microsatellites, potentially providing higher resolution to genetic analyses. This genomic resource may improve the accuracy of the categorical assignment tests previously mentioned, but also provide the level of detail required to perform continuous assignments, which directly infer the geographic origin of individuals. In continuous genetic assignment, each individual of known geographic origin is used as reference independently, rather than being pooled with other representatives of predefined groups. Models that combine genotype and sampling coordinates of these reference individuals are then created to assign coordinates of origin to other individuals (Yang et al. 2012; Guillot et al. 2016; Battey et al. 2020). This method presents two advantages in comparison to categorical assignments. First, continuous assignment does not require the presence of genetic structure among reference individuals, allowing use where patterns of genetic variability are continuous (e.g., isolation by distance scenarios). Second, the inferred origin is more geographically constrained, being represented by coordinates instead of membership to a predefined group that could span hundreds of square kilometers.

Our objectives were to assess genetic structure and the accuracy of genetic assignment tests for white‐tailed deer in the mid‐Atlantic region using recently available SNPs. Specifically, we predicted that SNPs would provide: (1) a more detailed description of genetic structure across a large spatial extent that was limited using microsatellites (Walter et al. 2024), (2) categorical assignment tests with more accurate placement of individuals into their cluster of origin compared to the results obtained with microsatellites, and (3) continuous assignment tests that correctly infer the geographic origin of individuals. Understanding the pattern of genetic structure and identifying an effective method of origin inference can help understand the underlying factors contributing to the routes of disease spread.

## 2 Materials and Methods

### 2.1 Sampling and Genotyping

We included 1920 wild white‐tailed deer samples from various tissues, collected in Maryland (MD), New York (NY), Ohio (OH), Pennsylvania (PA), and Virginia (VA) from 2014 to 2022. These samples were obtained from hunter‐harvested individuals (sampling coordinates from township centroid), roadkill and deer captured for other research studies (GPS sampling coordinates), and individuals obtained through routine disease surveillance by collaborating management agencies (sampling coordinates from county centroids or one‐mile grid centroid). Wild deer that were captured as part of separate research studies were collected in accordance with protocols approved by the Pennsylvania State University Institutional Animal Care and Use Committee (IACUC #'s: 47054, 201800026, and 202202225). We assumed that, for the majority of our samples, the sampling coordinates were close to the individuals' real origin, and if there were cases where this was not true (e.g., inaccurate sampling coordinates provided by hunters, or samples without coordinates assigned to county centroid), they would constitute a small portion of our samples.

We extracted genomic DNA following manufacturer's protocol (Qiagen DNeasy Blood and Tissue kit). For samples that were below the minimum DNA quantity of 17 ng/μl, we concentrated the DNA using ethanol precipitation following the protocol provided by Qiagen (https://www.qiagen.com/us/resources/faq/305). We submitted DNA samples to North American Genomics (Decatur, GA, USA) for genotyping using the Axiom OVSNP60 Genotyping Array (ThermoFisher), retaining samples that had a Dish Quality Control value of at least 0.82 (ThermoFisher Scientific 2024). We used PLINK v1.90b7 (www.cog‐genomics.org/plink/1.9/, Purcell et al. 2007; Chang et al. 2015), an open‐source toolset for genomic analysis, to perform further quality control with the following commands: *geno* 0.1 (i.e., delete markers that have more than 10% of missing data), *mind* 0.1 (i.e., delete individuals that have more than 10% of missing data), and *maf* 0.05 (i.e., delete markers that have a minor allele frequency < 5%). We used *indep‐pairwise* command to recursively remove SNPs based only on pairwise genotypic correlation within a sliding window, with the following parameters: window size in SNPs = 50, number of SNPs to shift the window at each step = 5, and *r* ^2^ = 0.5 (Viengkone et al. 2016). After quality control, our dataset was comprised of 1754 samples, representing 91.4% of samples submitted for genotyping, and 63,834 SNPs. We discarded five samples that did not have sampling location; therefore, our study included 1749 samples (hereafter referred to as *dataset*, Figure 1) from which *n* = 894 had GPS coordinates, *n* = 42 had sampling coordinates from one‐mile grid centroid, *n* = 579 had sampling coordinates from Township centroid, and *n* = 234 had sampling coordinates from County centroid.

**FIGURE 1:**
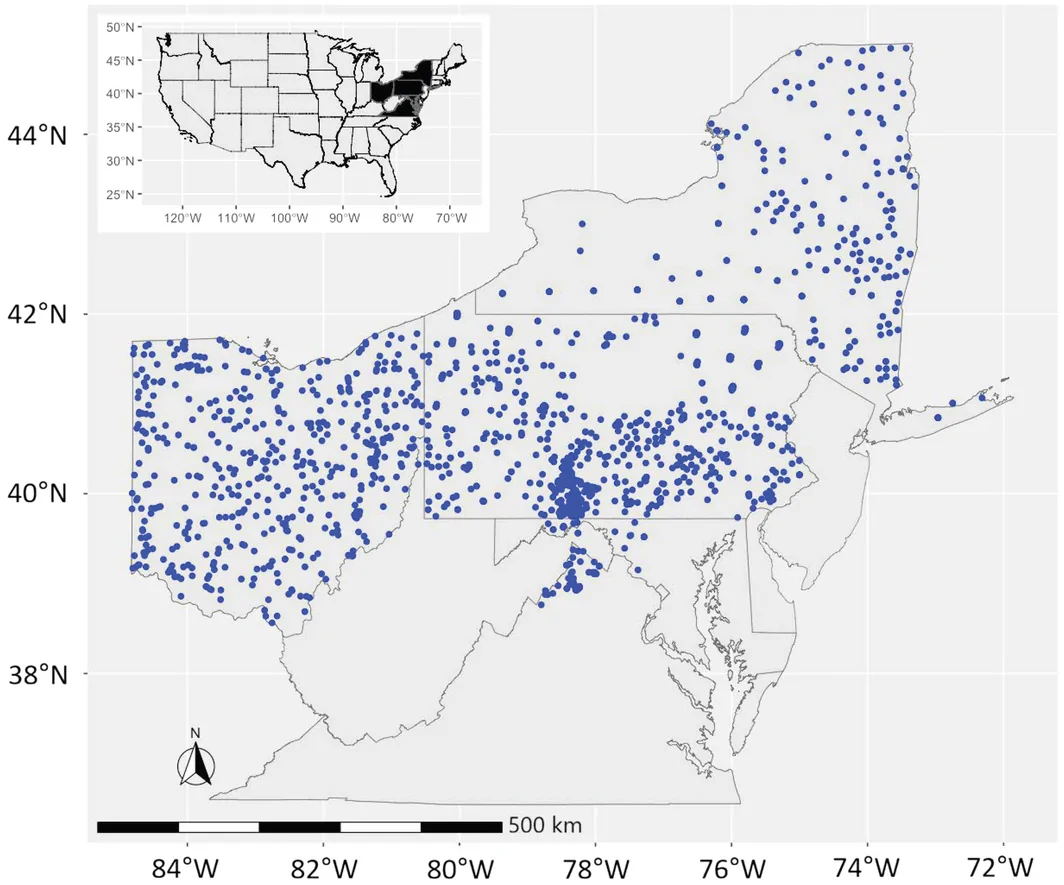
Map of our study area, with blue dots representing genotyped samples that we included in the analyses (*n* = 1749), collected from wild white‐tailed deer ( *Odocoileus virginianus* ). These samples were collected in the states of Ohio, Pennsylvania, New York, part of Maryland and part of Virginia.

Unless noted otherwise, we conducted all analyses described below in R software v4.4.2 (R Core Team 2024).

### 2.2 Geographic Distribution of Genetic Variability

We assessed global genetic diversity for the dataset by conducting a Principal Component Analysis (PCA) using PLINK, which by default extracts the top 20 principal components (PCs) of the variance‐standardized relationship matrix.

It is important to note that genetic structure is a hierarchical phenomenon, and different methods can pick up signals of genetic differentiation at different levels (Meirmans 2015). By applying different methods, we can achieve a higher level of confidence in the presence of particular barriers that are persistently inferred through different tests. We ran a Discriminant Analysis of Principal Components (DAPC, Jombart et al. 2010) using the ADEGENET package (Jombart 2008). Unlike PCA, which represents total genetic variation, DAPC focuses on capturing differences between predefined groups and minimizing intragroup variability by seeking synthetic variables (i.e., discriminant functions). To determine the number of groups present (*K*), we followed the DAPC tutorial (Jombart and Collins 2015) and used the *find.clusters* function, which transforms the raw data using PCA and then applies the *k*‐means algorithm, a multivariate clustering method, to infer the presence of clusters. *K*‐means does not use a population genetic model, and does not account for admixture (i.e., individuals having mixed ancestry). We knew from previous experience that different runs of the *find.clusters* function can show different number of clusters as the optimal solution. Therefore, we ran the function five times, in each case setting *K* = 1–20, and retaining the first 1000 PCs to reduce computational time, as we determined that this number of PCs already contained approximately 70% of cumulative variance (Figure S1a). We chose the *K* value that produced the lowest Bayesian Information Criterion as our optimal number of clusters. With this information we ran our DAPC using the dapc function, which also transforms the raw data using PCA. Once again we retained the first 1000 PCs. When running DAPC, retaining too many PCs can lead to overfitting (Jombart and Collins 2015). Note that, unlike *k*‐means, DAPC provides membership probabilities for each individual to each genetic cluster.

We used three different model‐based clustering approaches to explore the possibility of genetic structure in white‐tailed deer. Two of these methods use genotypic data exclusively, while the third method also incorporates the geographic coordinates of samples as priors for the clustering analysis. First, we conducted a STRUCTURE‐like analysis following the commands and tutorial developed by François (2016). This approach allows users to evaluate the presence of distinct genetic clusters in a dataset and estimate individuals' membership coefficients for each cluster without incorporating geographic coordinates. The analysis uses the sNMF algorithm implemented in the LEA package (Frichot and François 2015), which is conceptually similar to the clustering approach implemented in the widely used STRUCTURE software (Pritchard et al. 2000) but is implemented in R. We ran the analysis for *K* number of genetic clusters = 1–15, with five repetitions for each value of *K*, keeping the remaining parameters at their default values. To select the optimal number of genetic clusters in our dataset, we evaluated the cross‐entropy criterion as a function of *K*, choosing the *K* for which the cross‐entropy criterion was lowest (François 2016).

We also used fastStructure (Raj et al. 2014), a Python‐based algorithm which does not include geographic coordinates of samples as priors. We ran this analysis for *K* number of groups = 1–20, five repetitions per *K*, and using the default convergence criterion. A peculiarity of fastStructure is that the user also needs to select a model to generate population‐specific allele frequencies. These are the “simple prior” model, which is computationally faster but less likely to resolve subtle genetic structure, and the “logistic prior” model, which is recommended for cases of subtle genetic structure but might cause overfitting of the model, creating spurious clusters (Raj et al. 2014). We conducted the analysis for both available prior models with the parameters mentioned above; therefore, we obtained five outputs per *K* per model. For each model, we selected the best output to represent each *K* (i.e., the output with the highest marginal likelihood based on the five repetitions performed for that specific *K*), and used the chooseK.py script to determine the optimal number of clusters present in our data. Given the results of the logistic prior model, we decided to repeat the analysis for this model using five‐fold cross‐validation, to determine if we could obtain more clarity in the structure pattern by calculating cross‐validation error.

Finally, we evaluated genetic structure using the spatially explicit approach implemented in the package TESS3R (Caye et al. 2018, 2016; Martins et al. 2016). By incorporating geographic information of samples as priors, this method can outperform the previously mentioned nonspatial clustering algorithms in detecting subtle structure. We ran this analysis with *K* = 1–20, five repetitions per *K*, method = projected.ls, and all other parameters in their default values. We selected the value of *K* for which cross‐entropy reached a plateau as the optimal number of clusters present in our data (Caye et al. 2016; Frichot and François 2015).

We evaluated the geographic distribution of genetic clusters inferred with each of the three Bayesian clustering methods applied. We created a map showing spatial interpolation of membership values using the *plot* function from the TESS3R package (Caye et al. 2018, 2016). We applied “max” as the method to plot membership (i.e., each interpolated point is colored to represent the cluster contributing the most to membership), and FieldsKrigModel as the model to compute the interpolation surface, with a smoothness parameter of one. We created a palette of ten levels per color to represent membership to each cluster.

### 2.3 Categorical Assignments

We decided to use the groups obtained with the STRUCTURE‐like analysis in R as the basis to perform categorical assignments. The fastStructure and TESS3R clustering methods were either inconclusive or did not find a number of clusters that would be useful to approximate geographic origin of deer. For each sample, we knew which cluster it belonged to based on STRUCTURE‐like clustering analysis (i.e., the cluster for which they obtained the highest membership value). Our reasoning was to (1) use the best representatives of each cluster as reference individuals to represent the categories (i.e., genetic clusters), (2) assign all other individuals to the categories based on their similarity to reference individuals, and (3) evaluate if these assignments placed individuals in their correct cluster.

We used the package ASSIGNPOP (Chen et al. 2018), which performs cross‐validations to evaluate the baseline data, as a first step to assess the power of categorical assignment tests. To do so, we first ran ASSIGNPOP using only the best representatives of each cluster which were defined as individuals that obtained a membership of 0.6 or higher to any of the ten clusters inferred with STRUCTURE‐like analysis. This resulted in the following number of individuals for each cluster: *n*_cluster01_ = 37; *n*_cluster02_ = 124; *n*_cluster03_ = 107; *n*_cluster04_ = 50; *n*_cluster05_ = 64; *n*_cluster06_ = 55; *n*_cluster07_ = 67; *n*_cluster08_ = 22; *n*_cluster09_ = 40; *n*_cluster10_ = 74. We ran a Monte‐Carlo cross‐validation with these individuals, using the following settings: train.inds = 0.7, 0.8 and 0.9; train.loci = 0.75 and 1; loci.sample = “fst”; model = “svm,” and five iterations. This refers to the use of either 70%, 80%, or 90% of the individuals from each cluster as reference, while performing the assignment tests with the remaining 30%, 20%, or 10% of individuals per cluster, respectively. We also used either the 75% most informative loci (i.e., the ones with highest *F*_ST_ value) or all loci. From the multiple classification models available, we used support vector machine (model = “svm”) which is the default model, as we were unsure about the data distribution. For each combination of parameters, we ran five iterations (e.g., five runs where train.inds = 0.7 and train.loci = 1); therefore, we had a total of 30 runs.

After evaluating the baseline data and confirming the accuracy of assignments, we performed tests with the entire dataset, using as reference the previously mentioned individuals that obtained a membership of 0.6 or higher to any of the ten clusters inferred with STRUCTURE‐like analysis, and the rest as test individuals. Once again, we used the default support vector machine model to conduct the assignment tests.

### 2.4 Continuous Assignments

We tested two methods developed to assign geographic coordinates of origin to individuals, based solely on their genetic information. First, we used the Spatial Ancestry analysis (SPA) software (Yang et al. 2012), which uses reference individuals to model the allele frequencies at each SNP as a continuous two‐dimensional function of geographic coordinates, under the assumption that allele frequencies vary smoothly across geographic space. The method fits a surface for each SNP, describing how allele frequency varies across space, and by stacking this information from thousands of SNPs, it identifies the most likely geographic origin of a test genotype. By creating this two‐dimensional, rectangular area, SPA can potentially assign a sample to an origin that is not represented by any of the reference samples. We followed an iterative leave‐one‐out approach: all but one sample were used to build the model, and then we inferred the location of the test sample. We performed this analysis so that each sample would be tested once against all others. The SPA analysis allows users to specify how many origins are expected, one or two. For each individual, we ran the analysis to obtain a single location of origin (i.e., one set of coordinates) and two. To evaluate the accuracy of the results obtained with the single‐origin method, we projected the inferred coordinates and calculated the Euclidean distance between them and the sampling location coordinates. For the two‐origin method, we projected the inferred coordinates, calculated the midpoint between them, and calculated the Euclidean distance between this midpoint of origins and the sampling location.

Second, we used the supervised machine learning method LOCATOR (Battey et al. 2020), an analysis based on deep neural networks. Unlike SPA, LOCATOR does not assume any specific model of how allele frequencies vary across space. The method learns patterns in the training data, relating genotypes to locations, and uses those patterns to predict the geographic origin of new individuals. Before running LOCATOR, we used the *snp_fastImputeSimple* function of the BIGSNPR package (Privé et al. 2018) to replace missing values in genotypes and applied the “random” method (i.e., sampling based on allele frequencies). As we did with SPA, we followed an iterative leave‐one‐out approach to run LOCATOR, so that each sample would be tested once against all others. We then projected the inferred coordinates of origin and calculated the Euclidean distance between them and the sampling location.

## 3 Results

### 3.1 Geographic Distribution of Genetic Variability

The first two PCs of the PCA combined captured 38.9% of the total variance. The distribution pattern of samples in the PC space loosely reflected their geographic distribution (Figure 2). New York samples, which represent the northeast limit of our study area, were located mainly in the upper‐right corner of the PCA plot, while Ohio samples (west of our study area) congregated on the left side of the plot. Pennsylvania samples showed a high level of dispersion, while samples from Virginia and Maryland congregated in the lower‐right section of the PCA plot.

**FIGURE 2:**
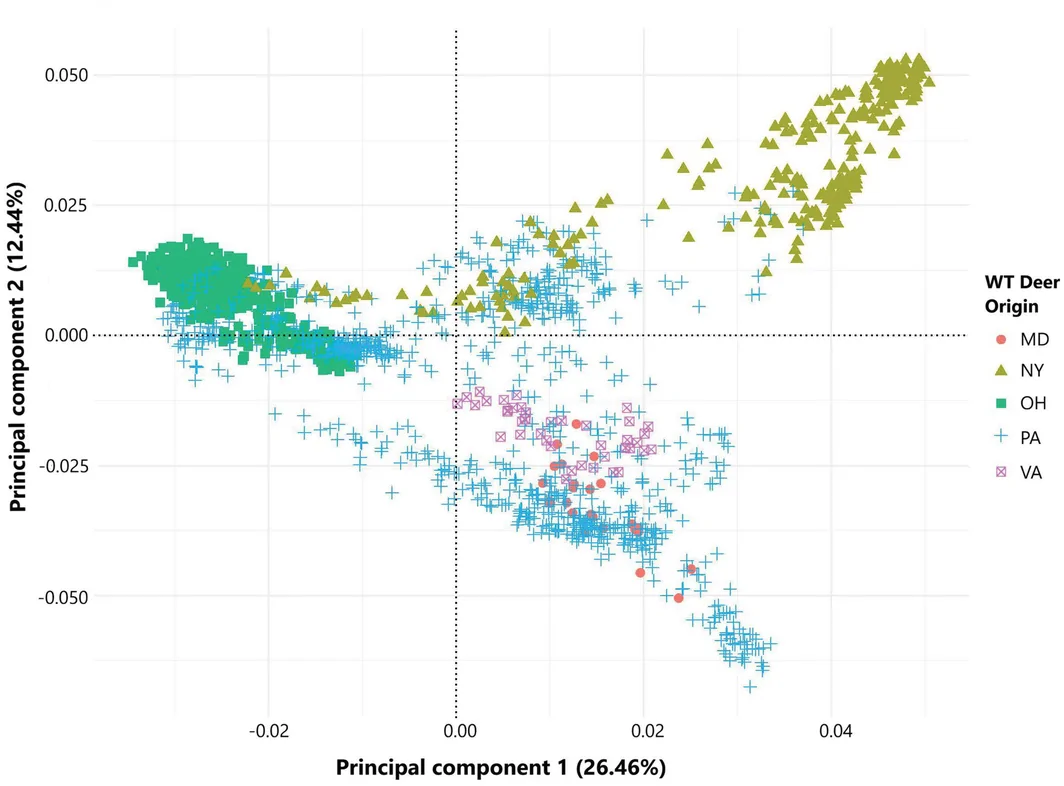
Results of the Principal Component Analysis conducted in PLINK with our white‐tailed deer samples collected in northern Maryland (MD), New York (NY), Ohio (OH), Pennsylvania (PA), and northern Virginia (VA).

The *k*‐means algorithm indicated the presence of 5–8 clusters (see Figure S1b for results indicating *K* = 5). Following a conservative approach, we chose *K* = 5 to represent the structure in our dataset. These clusters inferred with *k*‐means were distributed as follows (Figure S1c): Cluster 1 mainly in PA and OH, with a few samples in NY; Cluster 2 mainly in NY, with a few samples in PA; Cluster 3 only in Ohio; Cluster 4 in PA and MD; Cluster 5 mainly in PA, with representatives in all other states except OH. All individuals from VA were assigned to Cluster 5.

The distribution of clusters among states based on DAPC slightly differed from *k*‐means results, but the main pattern persisted. Figure 3 shows these clusters on the two discriminant functions retained, and the geographic distribution of samples that had at least 60% membership to a cluster. Once again, the pattern in the multivariate space resembled the geographic origin of samples. Cluster 3 (mainly in western OH) appears in the far left of discriminant function 1, neighboring Cluster 1 (rest of OH and western PA). Cluster 2 (mainly northeastern NY) appears in the bottom‐right area of the discriminant functions plot, while Cluster 4 (south‐central PA and part of MD) appears at the top of the plot.

**FIGURE 3:**
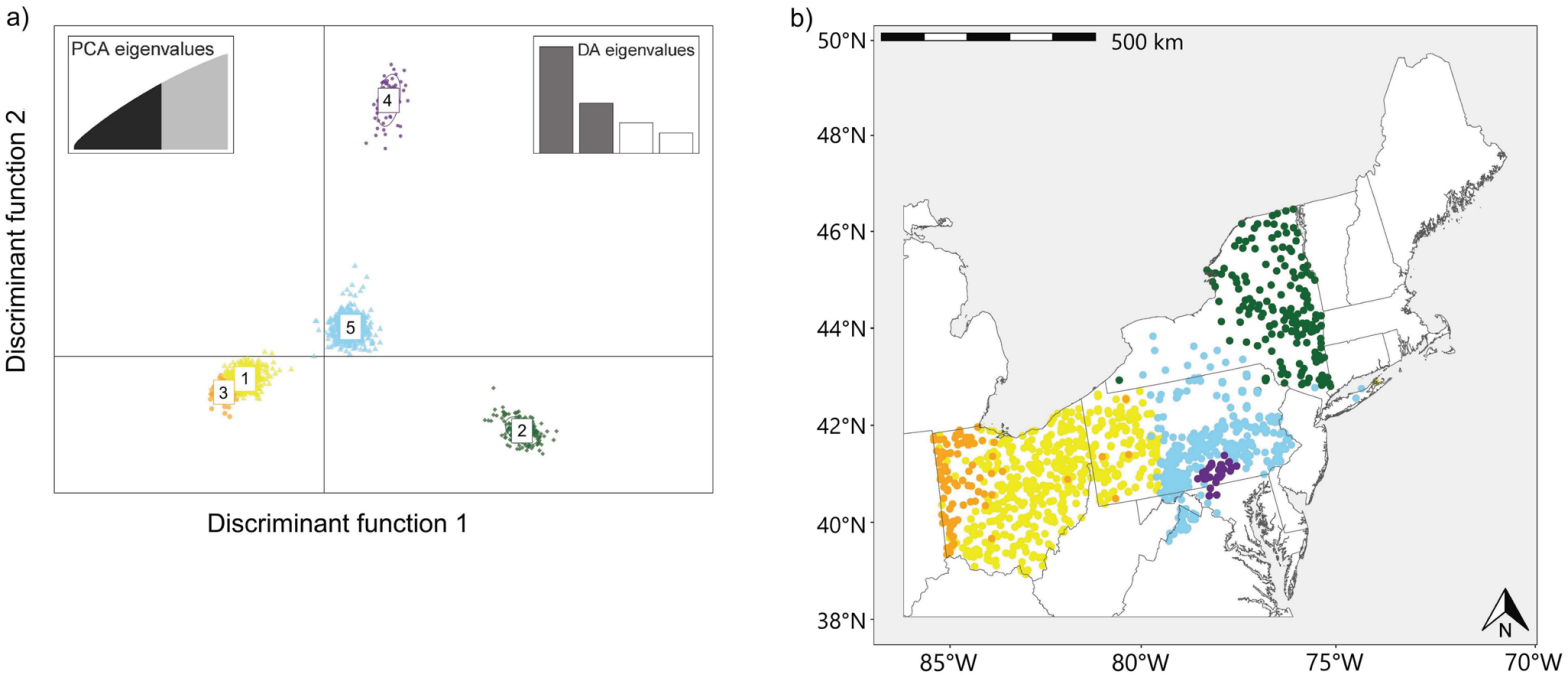
Results of the Discriminant Analysis of Principal Components conducted with our 1749 samples of white‐tailed deer. (a) The five inferred genetic clusters represented in two discriminant functions. Top‐left insert: Cumulative variance (y axis) explained by number of retained PCs (x axis); in black the 1000 PCs we retained to run DAPC. Top‐right insert: *F*‐statistic for the four discriminant functions obtained with DAPC; in gray the two discriminant functions we used for DAPC. (b) Geographic distribution of the five genetic clusters inferred. Circles represent only samples that had at least 60% membership to one of the clusters. Notice that the distribution of clusters among states differs slightly from the assignments obtained with the *k*‐means algorithm (Figure S1c).

The STRUCTURE‐like analysis indicated *K* = 10 as the most likely solution (Figure S2a). The map showing spatial interpolation of cluster memberships presents similarities with the map showing DAPC results, namely a genetic partition in OH, another one in PA along the area where the Appalachian Plateau meets the Valley and Ridge physiographic province, and another one creating a cluster in south‐central PA (Figure 4a,b).

**FIGURE 4:**
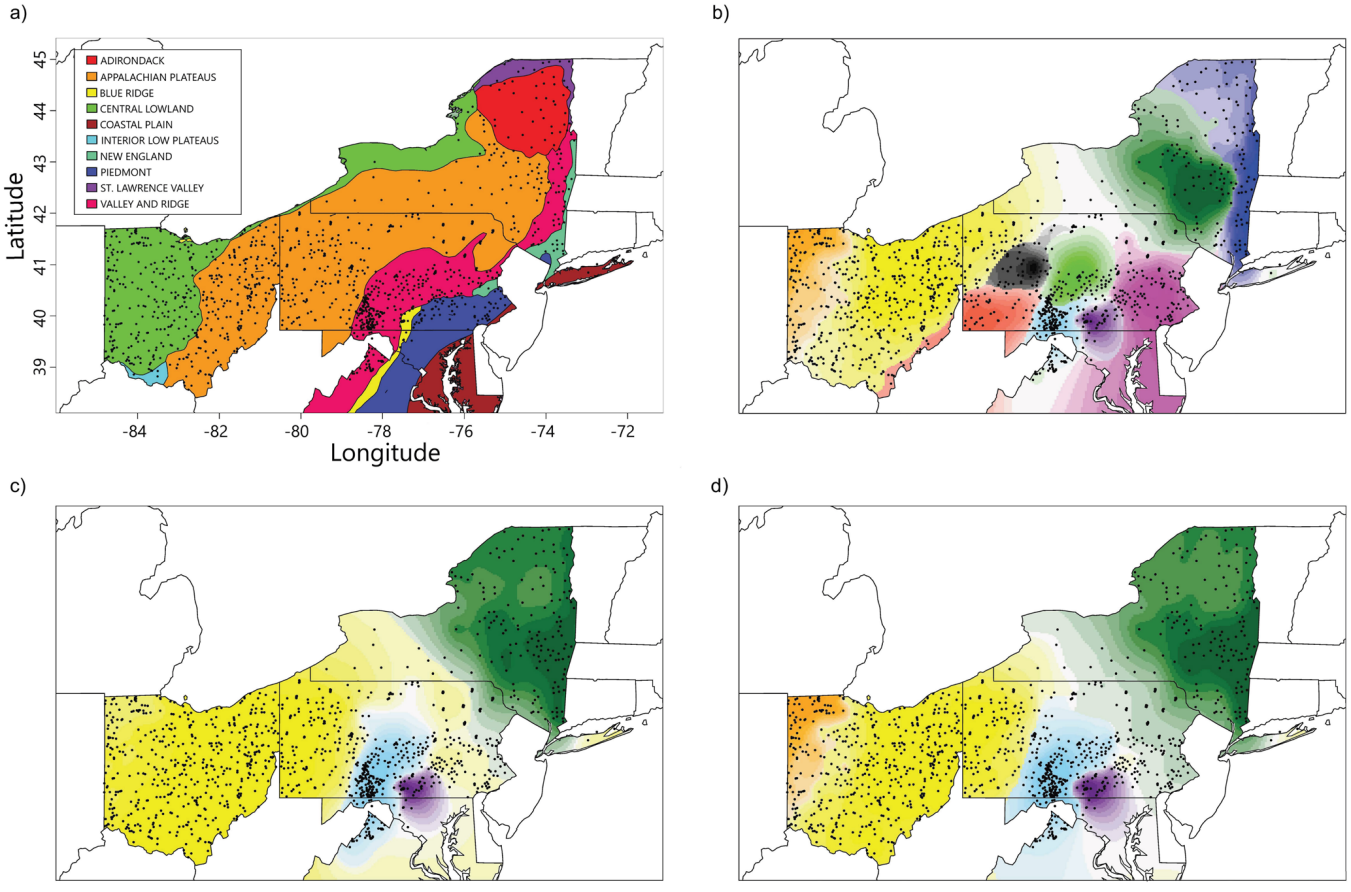
Genetic clustering results obtained with nonspatial and spatial Bayesian clustering algorithms using our white‐tailed deer samples. (a) Physiographic provinces present in our study area, with points representing samples, (b) STRUCTURE‐like analysis in R (optimal number of clusters = 10), (c) fastStructure, simple prior (optimal number of clusters = 4), (d) TESS3R for *K* = 5. The different colors in maps (b–d) represent interpolation of membership values to the inferred clusters (lighter shades equal lower membership to the cluster).

The fastStructure results using the simple prior model indicated that *K* = 4 was the model complexity that maximized marginal likelihood (see Figure S2b). The geographic borders of these genetic clusters coincided with borders detected with the STRUCTURE‐like analysis and DAPC (Figure 4c). The logistic prior model indicated *K* = 20 as the best model, but only one model component was used to explain structure in data. This means that even though the model allows up to 20 clusters, only one cluster carries most of the genetic signal across individuals, suggesting no meaningful structure under this prior. The runs showed a monotonic increase in marginal likelihood for *K* values higher than 5 (Figure S2c). Analyzing the cross‐validation error did not help to clarify the clustering pattern, as this error did not show a consistent trend of reduction with increasing *K* values (Figure S3). Therefore, we regarded the results obtained with this method as inconclusive.

The spatially explicit clustering approach implemented by TESS3R did not show a plateau in the cross‐entropy criterion vs. *K* plot (Figure S2d), and the model continued to gain support up to *K* = 20 (the maximum *K* tested). It is important to note, however, that for *K* values above five, individuals had values of membership < 0.5 to any of the clusters. This means that, by increasing the number of clusters beyond five, clusters become represented only by individuals with low membership. Therefore, we regarded the results obtained with this method as inconclusive. The interpolation showing spatial distribution of cluster membership on a map for the best *K* = 5 run returned a pattern similar to the one obtained with DAPC results: a genetic partition in OH, another one in PA along the area where the Appalachian Plateau meets the Valley and Ridge physiographic province, and another one creating a cluster in south‐central PA (Figure 4d).

### 3.2 Categorical Assignments

The Monte‐Carlo cross‐validation with the best representatives of each STRUCTURE‐like analysis cluster showed a 100% accuracy assigning individuals to their cluster for all five iterations, regardless of the proportion of individuals used as reference or the proportion of loci included (Table S1). As expected, the accuracy of assignments to most of the clusters decreased when we used the rest of our samples as test individuals. Average accuracy was 71%, and it ranged between 41% and 100% depending on the cluster (Table S2).

### 3.3 Continuous Assignments

We did not consider the analysis using SPA because, after analyzing 202 samples, the inferred locations were extremely far from the sampling locations. This was the case regardless of whether we inferred one or two origins (Figure S4). Using LOCATOR, the average distance between sampling location and inferred genetic origin was 29.9 km. This analysis also showed that 1435 samples (i.e., 82%) were assigned to a genetic origin less than 50 km from their sampling coordinates (Figure 5a). Among the remaining 314 samples, 156 (i.e., 49.7%) came from individuals that did not have exact sampling coordinates, which we assigned to the centroid of their county. Therefore, the LOCATOR‐inferred origin for these samples might in fact be closer to the real origin than we attributed. We detected eight individuals (four males and four females) whose inferred origin was > 150 km from their sampling location. In a closer inspection to the metadata provided by the agency that collected those samples we found that two of them, one male and one female (distance between inferred origin and sampling location was 247 km and 495 km, respectively), were collected for the same project and had the same sampling location. This could represent an error in the sampling location coordinates.

**FIGURE 5:**
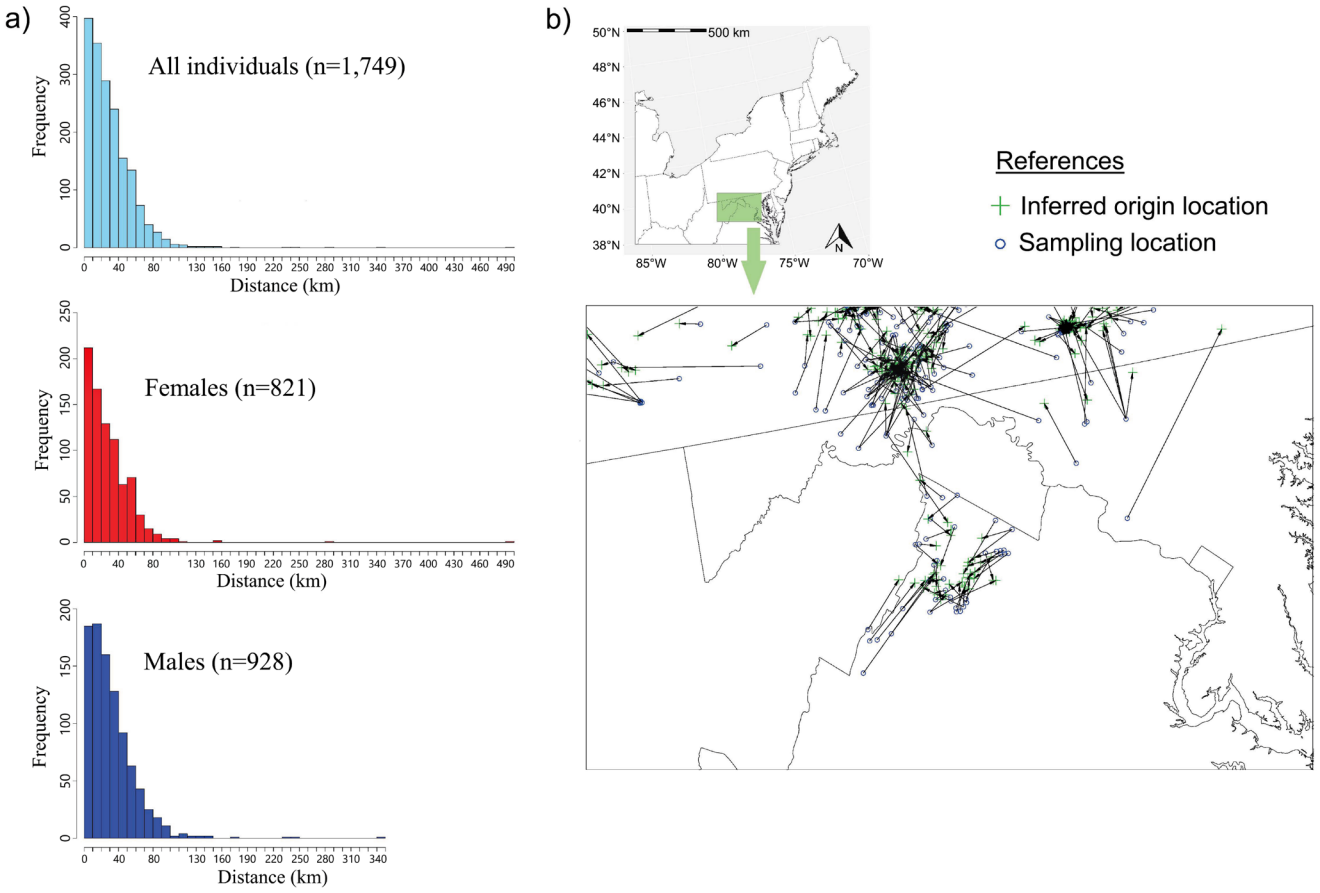
LOCATOR results for our white‐tailed deer samples. (a) Frequency histogram for all individuals, only females, and only males where the x axis represents the distance between the inferred point of origin and the true sampling origin. A total of 1435 samples (i.e., 82%) were assigned an origin less than 50 km from their sampling coordinates. (b) Example showing a fraction of our study area (southern Pennsylvania, part of Maryland and part of Virginia) and arrows representing distance between sampling locations (blue circles) and inferred genetic origin (green crosses).

## 4 Discussion

Numerous studies have evaluated deer movements with data from global positioning system or very high frequency collars in order to better understand the spatial dynamics involved in CWD spread (Oyer et al. 2007; Evans et al. 2016; Edmunds et al. 2018; Gilbertson et al. 2025). The detailed movement data obtained from such studies can inform the delineation of CWD management units, helping to avoid the creation of arbitrary boundaries (Jennelle et al. 2022); however, these approaches are limited to the areas in which studies were conducted, not statewide as is often desired. While genetic analyses are unable to provide the same level of resolution to assess animal movements, they are the best option available to trace back the origin of an individual while also assisting in the delineation of management units across states or regions. Regarding the latter, it is assumed that population genetic structure limits disease spread, because both gene flow and disease transmission depend on animal movements (Kelly et al. 2010).

Many wildlife management plans require information about animal density, and accurate density estimates depend on defining spatial units that correspond to the distribution and movements of the population being monitored (Goode et al. 2014; Parmenter et al. 2003). If management units do not encompass the area occupied by a population or subpopulation, changes in abundance may be difficult to detect because animals can redistribute within the broader population while moving across artificial management boundaries. For example, an action intended to increase deer abundance within a management unit could appear ineffective if deer respond by moving into adjacent areas outside the unit, even though the action increased abundance within the broader population. Delineating demographic units based on genetic structure can therefore help define spatial units that better reflect population structure and movement, improving the interpretation of density estimates and the evaluation of management actions. It can also help identify areas within which CWD may be capable of spreading through natural animal movements.

We found evidence of genetic structure in our study area; however, the number of clusters inferred varied depending on the method applied. This is not uncommon because, as previously mentioned, different methods can pick up signals of differentiation at different levels of the hierarchical genetic structure. It is important to note that our clustering results inferring higher *K* represent subdivisions of larger clusters inferred at lower *K*, with little variation in the location of barriers. For example, the barriers surrounding the light blue cluster inferred with fastStructure (Figure 4c) and TESS3R (Figure 4d) are also present in the STRUCTURE‐like analysis results (Figure 4b), even though this cluster gets defined as two clusters. Similarly, the barrier surrounding the purple cluster in southern PA or the barrier separating western OH from the rest were recovered in different structure configurations, even with DAPC. However, we found that applying a Bayesian clustering algorithm that includes coordinates as priors (TESS3R) lead to overestimating the number of genetic clusters in our dataset. The incorporation of sample coordinates in spatial clustering approaches means that these methods can outperform nonspatial approaches, detecting low levels of population differentiation (Guillot et al. 2005), but they can also overestimate the number of clusters (Chen et al. 2007). In our study, the spatial Bayesian clustering approach showed that the model continued to gain support up to the maximum number of clusters tested, but in scenarios where *K* > 5, individual membership to clusters was below 0.5. These results are similar to those reported by Smith et al. (2014), where authors found that a nonspatial Bayesian clustering method detected one population in the area of study, while a spatial approach found *K* = 3, although individuals had low membership to these clusters. Therefore, authors concluded there was no convincing evidence of genetic structure.

In a previous assessment of genetic structure using microsatellites (Walter et al. 2024), we only detected two clusters when using nonspatial Bayesian clustering methods. The fact that we inferred four (Figure 4c) and ten clusters (Figure 4b) in the present study, also using nonspatial Bayesian clustering methods, can be attributed to the higher resolution power that SNPs provide. Even DAPC showed clusters consistent with those found with Bayesian clustering algorithms, despite being a multivariate non‐model based analysis. In the previous study with microsatellites using a spatial Bayesian clustering method, a pattern of genetic structure was defined which was highly consistent with the clustering results with SNPs, despite not using the exact same samples or sample size in both assessments.

We decided to use the ten clusters obtained with the STRUCTURE‐like analysis as substrate for categorical assignments, as this higher number of clusters would provide more resolution when attempting to infer the geographic origin of individuals. The accuracy in inferring the geographic extent of these clusters has limitations, especially in under‐sampled areas like north‐central PA, as it derives from an interpolation of membership values over areas with no samples. Nonetheless, the borders of these clusters seem to match the presence of certain landscape features, which could be acting as barriers to gene flow (Figure S5). In PA, the western border of the light blue and light green clusters (Figure 4b) could be explained by the Allegheny Front, a topographic feature that marks the western boundary of the Valley and Ridge physiographic province (Figure 4a). In eastern NY, the border between the blue and green clusters correlates with the Hudson river and Interstate‐87. In western OH, the barrier between the orange and yellow clusters, also found in previous microsatellite analysis (Walter et al. 2024), could be a result of Interstate‐75. The purple cluster in southern PA‐northern MD is surrounded by interstates 70, 81 and 83, and the borders of the fuchsia cluster could be explained, at least in part, by the Susquehanna river.

A particular landscape feature can create a strong barrier for gene flow in part of its extent, and not in others. This is evident between the blue and dark green clusters in NY: the feature creating this division has a strong effect in the central part of the state, evidenced by the high contrasting membership values over short distances (i.e., dark shade of green and blue on opposite sides of the barrier). However, the effect disappears to the north of the state, which contains more genetically admixed individuals. These findings have implications for predicting the spread of CWD in different areas. In the case of NY, an introduction of CWD from PA could naturally reach the area represented in dark green (Figure 4b), but the spread further east seems less probable unless it is done through the northern part of the state, where the barrier between blue and green clusters appears weaker. This is particularly important as a recent detection of CWD in a captive facility in Wayne county, PA (which borders NY) led to the creation of Disease Management Area 10 in April 2025. In the case of OH, recent detections (years 2020–2023) led to the creation of a disease surveillance area which has since expanded to include the counties of Wyandot, Hardin, Marion, and eastern part of Allen (Ohio Department of Natural Resources 2025). All these detections were located east of Interstate 75, which seems to act as a barrier for deer gene flow and potentially their movements. Based on the genetic structure results we obtained, it seems unlikely that CWD will naturally expand further west from these infections.

The high level of genetic structuring in Pennsylvania, previously found with microsatellites and now confirmed through the SNP analyses, can also provide additional details on CWD spread dynamics in the state. There have been multiple instances of CWD‐positive deer found in wild deer from Pennsylvania (Pennsylvania Game Commission 2025), but given the genetic structure we found, it seems unlikely that these spatially disparate detections in wild deer are a product of natural CWD expansion due to animal movements, as this would imply multiple events of infected deer crossing the apparent barriers to gene flow. Under this scenario, more research to understand potential human‐mediated spread of CWD is warranted.

Categorical assignments of deer to the ten inferred clusters had a 100% accuracy when we tested the method only with individuals that had previously been assigned a membership value of at least 0.6 to a cluster, with 0.6 being a relatively low threshold. Using these individuals as reference and performing assignments with the remaining samples had much lower accuracy, due to the presence of many admixed individuals (highest membership of only 0.2–0.5 to a cluster). Therefore, we consider this method to be unsuitable for inferring geographic origin of wild deer. Categorical assignments have the potential to be useful when determining a captive or wild origin of deer, as done previously with microsatellites (Miller and Walter 2020; Walter et al. 2024). In that scenario, continuous assignments are not appropriate to infer individuals' origin, as these methods are based on spatial patterns of genetic variability. While a method like LOCATOR is more flexible and able to learn nonlinear relationships between genotype and geographic location, the nonnatural relationships present in captive facilities (i.e., animals brought in from different stocks and across state boundaries may be highly differentiated genetically but now coexisting in close proximity) will introduce confounding patterns that affect the accuracy of the assignments when captive deer are included as reference individuals. A different scenario, where captive deer are not used as references but as test individuals, is also problematic because their genotype will not fit the natural pattern of variability. These deer would be assigned to a location somewhere in the area represented by the reference individuals, even though their genetic ancestry might not be present in this area.

Regarding our evaluation of continuous assignment tools, SPA showed a high level of inaccuracy to infer geographic origin of deer, which could be attributed to the presence of genetic structure. The method implemented in SPA requires a monotonic function to model how allele frequencies change with geographic position, and therefore cannot capture instances of multiple peaks in the allele frequency surface (Yang et al. 2012; Qin et al. 2022). On the other hand, LOCATOR was able to assign most of the samples to a location < 50 km from their sampling point, and the average distance between inferred origin and sampling coordinates was 29.9 km. While a rigorous evaluation of LOCATOR's performance should assess the distance between inferred and real geographic origin, the latter is not easily available for wild animals. Therefore, we were constrained to evaluate the distance between inferred origin and sampling location, which does not necessarily match due to natural dispersal of individuals. Even if we had access to information about the real geographic origin of each deer, a certain degree of error is to be expected when assigning genetic origin in a large, vagile species such as white‐tailed deer.

Furthermore, the cumulative phenomenon of dispersion, where close relatives in each generation can disperse in different directions, combined with polyandry and polygyny, obscures the association between location and genotype. LOCATOR has recently been used to detect translocations of feral swine ( *Sus scrofa* ), using samples from the USA and Canada, and categorizing individuals as residents or translocated depending on whether they had an inferred origin location within 200 km of their sampling location (Giglio et al. 2024). This tool has also been used to infer the origin of white‐tailed deer in Arkansas (Chafin et al. 2021), with the LOCATOR analysis being repeated 100 times per deer to assess variability among predictions for the same individual. The results obtained cannot be directly compared to the ones presented in our study, as the geographic scale was much smaller (roughly 138 km^2^ vs. 380 km^2^), the number of SNPs was 5,000, and the results focused on individuals for which variability among LOCATOR predictions was low (*N* = 110 or 264 depending on the threshold used). Other studies implementing different tools for continuous assignments found greater distance between sampling coordinates and genetic origin: median of 188.7 km for Spanish cedar ( *Cedrela odorata* , Finch et al. 2020), mean of 80.45 –707.67 km depending on biome for jaguars ( *Panthera onca* , Zenato Lazzari et al. 2025).

A caveat of our study is the lack of precise sampling coordinates for some of the deer included: 234 deer (i.e., 13.4%) had a sampling location corresponding to County centroid, while 579 deer (i.e., 33.1%) had a sampling location corresponding to Township centroid. Given that we evaluated the precision of LOCATOR results based on the distance between inferred origin and sampling location, it is possible that the accuracy of continuous assignments for these samples was in fact better than attributed. The LOCATOR tool allowed us to find potential errors in our dataset (i.e., individuals that were assigned an origin far too distant to their sampling location), and therefore we can eliminate these deer from the reference dataset used in future analyses. State agencies may attempt to get more detail on the origin of those deer because they likely did not come from their reported locations, and they could represent wrong reported sampling origin or deer that escaped from captive facilities. Female white‐tailed deer are assumed to be more philopatric than males, but differences in dispersal behavior have been described in separate regions (Lutz et al. 2015; Jennelle et al. 2022). The general pattern of distance between inferred origin and sampling location shown as histograms in Figure 5 did not hint to one sex being more frequently assigned farther from sampling location.

Taking into account the spatial extent covered in our study area, the lack of information about the real origin of individuals, and the characteristics of white‐tailed deer such as natal dispersal and overlapping generations, we consider the continuous assignments obtained with LOCATOR to be highly accurate (average 29.9 km, most deer assigned an origin < 50 km from sampling location), and therefore useful for inferring the geographic origin of wild white‐tailed deer. Providing researchers or agencies with locations and genotypes of samples collected over large spatial extents may provide a valuable resource to identify the true geographic origin of wild deer.

## Author Contributions

**Alberto F. Fameli:** conceptualization (equal), data curation (equal), formal analysis (lead), investigation (equal), methodology (equal), software (lead), visualization (lead), writing – original draft (lead), writing – review and editing (lead). **Jessie E. Edson:** conceptualization (supporting), data curation (lead), methodology (equal), resources (equal), writing – review and editing (equal). **Krysten L. Schuler:** conceptualization (equal), investigation (equal), methodology (equal), resources (equal), writing – review and editing (equal). **W. David Walter:** conceptualization (equal), funding acquisition (lead), project administration (lead), resources (equal), supervision (lead), writing – review and editing (equal).

## Funding

This work was supported by funding from Pennsylvania Game Commission (Research Project Numbers 27 and 39) and U.S. Geological Survey, Ecosystem Mission Area Cyclical Funding FY 20–22 (RWO 96). Additional samples were provided by D. Diefenbach from funding provided by the Pennsylvania Game Commission under a separate agreement (Research Project No. 23). Extra funds for materials were provided by the Virginia Department of Game and Inland Fisheries.

## Conflicts of Interest

The authors declare no conflicts of interest.

## Supporting information


**Figure S1:** Results of the *k*‐means algorithm. (a) Percentage of cumulative variance vs. principal components (PCs) obtained when using the *find. clusters* function. (b) The *k*‐means algorithm was run five times assuming 1–20 clusters (*K*), and the Bayesian Information Criterion (BIC) showed *K* = 5–8 as the optimal clustering solution. We followed a conservative approach and chose *K* = 5 (shown in figure). (c) Distribution of the five inferred genetic clusters (C1–C5) among the five states represented in our study: Pennsylvania (“PA”), New York (“NY”), Ohio (“OH”), Maryland (“MD”) and Virginia (“VA”).
**Figure S2:** Plots used to determine the optimal number of clusters (*K*) present in our dataset of 1749 white‐tailed deer samples, genotyped for 63,834 markers. (a) Cross‐entropy vs. *K* obtained in the STRUCTURE‐like analysis in R (selected *K* = 10); (b) marginal likelihood vs. *K* in the fastStructure analysis, using the simple prior model (selected *K* = 4); (c) marginal likelihood vs. *K* in the fastStructure analysis, using the logistic prior model (no best *K* selected); (d) cross‐validation score vs. *K* obtained in the TESS3R analysis (no best *K* selected).
**Figure S3:** Results of fastStructure runs using the logistic prior model. While the marginal likelihood showed a maximum value for the 20 clusters model (Figure S2c), the cross‐validation error (CV) did not show a consistent downward trend as the number of clusters (*K*) increased, indicating limited improvement in predictive accuracy with increased model complexity.
**Figure S4:** Results of the spatial ancestry analysis (SPA) for the first 202 samples tested. The origin inferred for these samples (black dots) was obviously inaccurate whether we used (a) single‐origin model or (b) a midpoint between two origin locations inferred with the two‐origin model.
**Figure S5:** Results STRUCTURE‐like analysis and landscape features that could be explaining the pattern of genetic structure. The different colors represent interpolation of membership values to the ten inferred clusters (lighter shades equal lower membership to the cluster). (a) Red lines represent water bodies with a stream order value higher than five (downloaded from https://www.epa.gov/waterdata/nhdplus‐national‐data). (b) Red lines represent primary roads, obtained from the R package TIGRIS (Walker 2025).
**Table S1:** Results of the Monte‐Carlo cross‐validation performed for our categorical assignment tests using ASSIGNPOP. At this stage, we only included white‐tailed deer that had a membership of at least 0.6 to any of the ten clusters inferred in the STRUCTURE‐like analysis. We ran five iterations for each combination of parameters, which included proportion of reference individuals for each cluster (0.7, 0.8, or 0.9) and proportion of loci used (0.75 with highest Fst, or all loci).
**Table S2:** Results of the categorical assignments conducted with ASSIGNPOP, using as reference the individuals that had a membership of at least 0.6 to any of the ten clusters inferred in the STRUCTURE‐like clustering analysis. Inset figure: map showing geographic extent of each of the ten clusters, as shown in Figure 4b. Points represent samples, and the different colors show interpolation of membership values to the inferred clusters (lighter shades equal lower membership to the cluster). Each row shows test individuals and the cluster they belong to, and columns indicate the cluster they were assigned to during categorical assignment tests using ASSIGNPOP.

## Data Availability

Data will be permanently archived at Zenodo under DOI https://doi.org/10.5281/zenodo.19712287.
