# Supplementary material for: Inferring Geographic Origin of White‐Tailed Deer (Odocoileus virginianus) Using Single Nucleotide Polymorphisms in a Region Experiencing Long‐Term Effects of Chronic Wasting Disease
Source: Ecol Evol. 2026 Oct 9;16(10):e74454. doi: 10.1002/ece3.74454 (PMC13650593; doi:10.1002/ece3.74454)
Supplement: Supplementary file 1 [file ECE3-16-e74454-s001.docx]

**Supplementary material**

**Inferring geographic origin of white-tailed deer (*Odocoileus virginianus*) using single nucleotide polymorphisms in a region experiencing long-term effects of chronic wasting disease**

Alberto F. Fameli^1,2^ (alberto.fameli@wsu.edu), Jessie E. Edson^1^ (jee5023@psu.edu), Krysten L. Schuler^3^ (ks833@cornell.edu), and W. David Walter^1,2^ (wdavid.walter@wsu.edu).

^1^Department of Ecosystem Science and Management, The Pennsylvania State University, University Park, Pennsylvania, USA.

^2^Veterinary Microbiology and Pathology, Washington State University, Pullman, Washington, USA.

^3^Cornell Wildlife Health Lab, New York State Wildlife Health Program, Ithaca, New York, USA.

**Correspondence:** Alberto F. Fameli (alberto.fameli@wsu.edu). ORCID: 0000-0001-6566-405X

**Figure S1.** Results of the k-means algorithm. (a) Percentage of cumulative variance vs principal components (PCs) obtained when using the *find.clusters* function. (b) The k-means algorithm was run five times assuming 1─20 clusters (K), and the Bayesian Information Criterion (BIC) showed K=5─8 as the optimal clustering solution. We followed a conservative approach and chose K=5 (shown in figure). (c) Distribution of the five inferred genetic clusters (C1─C5) among the five states represented in our study: Pennsylvania (“PA”), New York (“NY”), Ohio (“OH”), Maryland (“MD”) and Virginia (“VA”).

**Figure S2.** Plots used to determine the optimal number of clusters (K) present in our dataset of 1,749 white-tailed deer samples, genotyped for 63,834 markers. (a) Cross-entropy vs K obtained in the STRUCTURE-like analysis in R (selected K=10); (b) marginal likelihood vs K in the fastStructure analysis, using the simple prior model (selected K=4); (c) marginal likelihood vs K in the fastStructure analysis, using the logistic prior model (no best K selected); (d) cross-validation score vs K obtained in the TESS3R analysis (no best K selected).

**Figure S3.** Results of fastStructure runs using the logistic prior model. While the marginal likelihood showed a maximum value for the 20 clusters model (Supplementary Figure S2c), the cross-validation error (CV) did not show a consistent downward trend as the number of clusters (K) increased, indicating limited improvement in predictive accuracy with increased model complexity.

**Figure S4.** Results of the spatial ancestry analysis (SPA) for the first 202 samples tested. The origin inferred for these samples (black dots) was obviously inaccurate whether we used (a) single-origin model or (b) a midpoint between two origin locations inferred with the two-origin model.

**Figure S5.** Results STRUCTURE-like analysis and landscape features that could be explaining the pattern of genetic structure. The different colors represent interpolation of membership values to the ten inferred clusters (lighter shades equal lower membership to the cluster). (a) Red lines represent water bodies with a stream order value higher than five (downloaded from <https://www.epa.gov/waterdata/nhdplus-national-data>). (b) Red lines represent primary roads, obtained from the R package TIGRIS (Walker 2025).

| Proportion of reference individuals | Loci | Iteration | Assignment accuracy rate | | | | | | | | | | |
| --- | --- | --- | --- | --- | --- | --- | --- | --- | --- | --- | --- | --- | --- |
|  |  |  | Cluster  01 | Cluster 02 | Cluster 03 | Cluster 04 | Cluster 05 | Cluster 06 | Cluster 07 | Cluster 08 | Cluster 09 | Cluster 10 | Overall |
| 0.7 | 0.75 | 1 | 1 | 1 | 1 | 1 | 1 | 1 | 1 | 1 | 1 | 1 | 1 |
| 0.7 | 0.75 | 2 | 1 | 1 | 1 | 1 | 1 | 1 | 1 | 1 | 1 | 1 | 1 |
| 0.7 | 0.75 | 3 | 1 | 1 | 1 | 1 | 1 | 1 | 1 | 1 | 1 | 1 | 1 |
| 0.7 | 0.75 | 4 | 1 | 1 | 1 | 1 | 1 | 1 | 1 | 1 | 1 | 1 | 1 |
| 0.7 | 0.75 | 5 | 1 | 1 | 1 | 1 | 1 | 1 | 1 | 1 | 1 | 1 | 1 |
| 0.7 | 1 | 1 | 1 | 1 | 1 | 1 | 1 | 1 | 1 | 1 | 1 | 1 | 1 |
| 0.7 | 1 | 2 | 1 | 1 | 1 | 1 | 1 | 1 | 1 | 1 | 1 | 1 | 1 |
| 0.7 | 1 | 3 | 1 | 1 | 1 | 1 | 1 | 1 | 1 | 1 | 1 | 1 | 1 |
| 0.7 | 1 | 4 | 1 | 1 | 1 | 1 | 1 | 1 | 1 | 1 | 1 | 1 | 1 |
| 0.7 | 1 | 5 | 1 | 1 | 1 | 1 | 1 | 1 | 1 | 1 | 1 | 1 | 1 |
| 0.8 | 0.75 | 1 | 1 | 1 | 1 | 1 | 1 | 1 | 1 | 1 | 1 | 1 | 1 |
| 0.8 | 0.75 | 2 | 1 | 1 | 1 | 1 | 1 | 1 | 1 | 1 | 1 | 1 | 1 |
| 0.8 | 0.75 | 3 | 1 | 1 | 1 | 1 | 1 | 1 | 1 | 1 | 1 | 1 | 1 |
| 0.8 | 0.75 | 4 | 1 | 1 | 1 | 1 | 1 | 1 | 1 | 1 | 1 | 1 | 1 |
| 0.8 | 0.75 | 5 | 1 | 1 | 1 | 1 | 1 | 1 | 1 | 1 | 1 | 1 | 1 |
| 0.8 | 1 | 1 | 1 | 1 | 1 | 1 | 1 | 1 | 1 | 1 | 1 | 1 | 1 |
| 0.8 | 1 | 2 | 1 | 1 | 1 | 1 | 1 | 1 | 1 | 1 | 1 | 1 | 1 |
| 0.8 | 1 | 3 | 1 | 1 | 1 | 1 | 1 | 1 | 1 | 1 | 1 | 1 | 1 |
| 0.8 | 1 | 4 | 1 | 1 | 1 | 1 | 1 | 1 | 1 | 1 | 1 | 1 | 1 |
| 0.8 | 1 | 5 | 1 | 1 | 1 | 1 | 1 | 1 | 1 | 1 | 1 | 1 | 1 |
| 0.9 | 0.75 | 1 | 1 | 1 | 1 | 1 | 1 | 1 | 1 | 1 | 1 | 1 | 1 |
| 0.9 | 0.75 | 2 | 1 | 1 | 1 | 1 | 1 | 1 | 1 | 1 | 1 | 1 | 1 |
| 0.9 | 0.75 | 3 | 1 | 1 | 1 | 1 | 1 | 1 | 1 | 1 | 1 | 1 | 1 |
| 0.9 | 0.75 | 4 | 1 | 1 | 1 | 1 | 1 | 1 | 1 | 1 | 1 | 1 | 1 |
| 0.9 | 0.75 | 5 | 1 | 1 | 1 | 1 | 1 | 1 | 1 | 1 | 1 | 1 | 1 |
| 0.9 | 1 | 1 | 1 | 1 | 1 | 1 | 1 | 1 | 1 | 1 | 1 | 1 | 1 |
| 0.9 | 1 | 2 | 1 | 1 | 1 | 1 | 1 | 1 | 1 | 1 | 1 | 1 | 1 |
| 0.9 | 1 | 3 | 1 | 1 | 1 | 1 | 1 | 1 | 1 | 1 | 1 | 1 | 1 |
| 0.9 | 1 | 4 | 1 | 1 | 1 | 1 | 1 | 1 | 1 | 1 | 1 | 1 | 1 |
| 0.9 | 1 | 5 | 1 | 1 | 1 | 1 | 1 | 1 | 1 | 1 | 1 | 1 | 1 |

**Table S1.** Results of the Monte-Carlo cross-validation performed for our categorical assignment tests using ASSIGNPOP. At this stage, we only included white-tailed deer that had a membership of at least 0.6 to any of the ten clusters inferred in the STRUCTURE-like analysis. We ran five iterations for each combination of parameters, which included proportion of reference individuals for each cluster (0.7, 0.8 or 0.9) and proportion of loci used (0.75 with highest Fst, or all loci).

**Table S2.** Results of the categorical assignments conducted with ASSIGNPOP, using as reference the individuals that had a membership of at least 0.6 to any of the ten clusters inferred in the STRUCTURE-like clustering analysis. Inset figure: map showing geographic extent of each of the ten clusters, as shown in Figure 4b. Points represent samples, and the different colors show interpolation of membership values to the inferred clusters (lighter shades equal lower membership to the cluster). Each row shows test individuals and the cluster they belong to, and columns indicate the cluster they were assigned to during categorical assignment tests using ASSIGNPOP.

|  | | **Categorical assignments** | | | | | | | | | |  |
| --- | --- | --- | --- | --- | --- | --- | --- | --- | --- | --- | --- | --- |
|  |  | Cluster 01 | Cluster 02 | Cluster 03 | Cluster 04 | Cluster 05 | Cluster 06 | Cluster 07 | Cluster 08 | Cluster 09 | Cluster 10 | Proportion of accurate assignments |
| **Membership in clustering analysis** | Cluster 01 | 107 | 44 | 0 | 5 | 0 | 0 | 0 | 0 | 0 | 0 | 0.69 |
|  | Cluster 02 | 0 | 332 | 0 | 1 | 0 | 0 | 0 | 0 | 0 | 0 | 1 |
|  | Cluster 03 | 0 | 0 | 128 | 0 | 0 | 0 | 0 | 0 | 0 | 0 | 1 |
|  | Cluster 04 | 0 | 0 | 0 | 96 | 0 | 0 | 0 | 0 | 0 | 1 | 0.99 |
|  | Cluster 05 | 0 | 12 | 0 | 0 | 42 | 0 | 0 | 0 | 0 | 0 | 0.78 |
|  | Cluster 06 | 0 | 0 | 0 | 1 | 0 | 25 | 0 | 0 | 0 | 26 | 0.48 |
|  | Cluster 07 | 0 | 0 | 2 | 3 | 0 | 0 | 9 | 0 | 0 | 0 | 0.64 |
|  | Cluster 08 | 0 | 27 | 15 | 0 | 0 | 0 | 0 | 29 | 0 | 0 | 0.41 |
|  | Cluster 09 | 2 | 4 | 38 | 10 | 0 | 0 | 0 | 0 | 49 | 0 | 0.48 |
|  | Cluster 10 | 0 | 4 | 0 | 33 | 0 | 0 | 0 | 0 | 0 | 64 | 0.63 |

**References**

Walker, Kyle. 2025. *Tigris: Load Census TIGER/Line Shapefiles*. V. version 2.2.1.9000. R Package. Released. https://github.com/walkerke/tigris.
